# Supplementary material for: Modeling glioblastoma heterogeneity as a dynamic network of cell states
Source: Mol Syst Biol. 2021 Sep 16;17(9):e10105. doi: 10.15252/msb.202010105 (PMC8444284; doi:10.15252/msb.202010105)
Supplement: Supplementary file 6 — Source Data for Figure 5 [file MSB-17-e10105-s004.zip › Figure5A_sourcedata/GSEA_3071/hallmarks_stateIV.GseaPreranked.1623742551702/HALLMARK_G2M_CHECKPOINT.html]

Details for gene set HALLMARK\_G2M\_CHECKPOINT[GSEA]

|  || Dataset | state53071 |
| Phenotype | NoPhenotypeAvailable |
| Upregulated in class | na\_neg |
| GeneSet | HALLMARK\_G2M\_CHECKPOINT |
| Enrichment Score (ES) | -0.53776026 |
| Normalized Enrichment Score (NES) | -2.9916825 |
| Nominal p-value | 0.0 |
| FDR q-value | 0.0 |
| FWER p-Value | 0.0 |
Table: GSEA Results Summary

  

Fig 1: Enrichment plot: HALLMARK\_G2M\_CHECKPOINT      
 Profile of the Running ES Score & Positions of GeneSet Members on the Rank Ordered List

  

| PROBE | GENE SYMBOL | GENE\_TITLE | RANK IN GENE LIST | RANK METRIC SCORE | RUNNING ES | CORE ENRICHMENT || 1 | MEIS2 |  |  | 32 | 2.004 | 0.0156 | No |
| 2 | MEIS1 |  |  | 269 | 1.107 | -0.0181 | No |
| 3 | FOXN3 |  |  | 348 | 0.977 | -0.0226 | No |
| 4 | CDKN1B |  |  | 374 | 0.939 | -0.0172 | No |
| 5 | SMARCC1 |  |  | 709 | 0.654 | -0.0748 | No |
| 6 | ATRX |  |  | 727 | 0.645 | -0.0711 | No |
| 7 | RPS6KA5 |  |  | 957 | 0.540 | -0.1096 | No |
| 8 | ARID4A |  |  | 1096 | 0.493 | -0.1309 | No |
| 9 | NUMA1 |  |  | 1338 | 0.431 | -0.1729 | No |
| 10 | MARCKS |  |  | 1713 | 0.348 | -0.2416 | No |
| 11 | SAP30 |  |  | 1914 | 0.311 | -0.2770 | No |
| 12 | BCL3 |  |  | 2125 | 0.280 | -0.3146 | No |
| 13 | ILF3 |  |  | 2159 | 0.274 | -0.3180 | No |
| 14 | LBR |  |  | 2271 | 0.260 | -0.3367 | No |
| 15 | PTTG1 |  |  | 2470 | -0.266 | -0.3721 | No |
| 16 | KIF5B |  |  | 2471 | -0.266 | -0.3692 | No |
| 17 | LMNB1 |  |  | 2487 | -0.268 | -0.3692 | No |
| 18 | CUL1 |  |  | 2617 | -0.285 | -0.3911 | No |
| 19 | CDC25B |  |  | 2671 | -0.292 | -0.3982 | No |
| 20 | CDC25A |  |  | 2753 | -0.301 | -0.4106 | No |
| 21 | RAD54L |  |  | 2855 | -0.313 | -0.4268 | No |
| 22 | NUP98 |  |  | 2920 | -0.321 | -0.4357 | No |
| 23 | EZH2 |  |  | 2946 | -0.324 | -0.4370 | No |
| 24 | NUP50 |  |  | 2954 | -0.326 | -0.4348 | No |
| 25 | SMC1A |  |  | 2968 | -0.328 | -0.4338 | No |
| 26 | SMC4 |  |  | 3035 | -0.337 | -0.4429 | No |
| 27 | SRSF10 |  |  | 3223 | -0.364 | -0.4752 | No |
| 28 | MCM2 |  |  | 3240 | -0.366 | -0.4743 | No |
| 29 | ORC5 |  |  | 3277 | -0.371 | -0.4772 | No |
| 30 | POLE |  |  | 3329 | -0.379 | -0.4830 | No |
| 31 | ODF2 |  |  | 3345 | -0.381 | -0.4818 | No |
| 32 | PRMT5 |  |  | 3442 | -0.397 | -0.4960 | No |
| 33 | TFDP1 |  |  | 3526 | -0.415 | -0.5076 | No |
| 34 | HIF1A |  |  | 3542 | -0.418 | -0.5060 | No |
| 35 | POLQ |  |  | 3600 | -0.429 | -0.5123 | No |
| 36 | TROAP |  |  | 3621 | -0.432 | -0.5115 | No |
| 37 | RAD23B |  |  | 3688 | -0.442 | -0.5195 | No |
| 38 | FANCC |  |  | 3689 | -0.442 | -0.5147 | No |
| 39 | NOLC1 |  |  | 3710 | -0.448 | -0.5137 | No |
| 40 | HSPA8 |  |  | 3720 | -0.449 | -0.5106 | No |
| 41 | CCNF |  |  | 3755 | -0.456 | -0.5122 | No |
| 42 | PLK4 |  |  | 3791 | -0.465 | -0.5139 | No |
| 43 | KIF22 |  |  | 3832 | -0.472 | -0.5166 | No |
| 44 | NOTCH2 |  |  | 3853 | -0.477 | -0.5152 | No |
| 45 | CDC27 |  |  | 3918 | -0.491 | -0.5223 | No |
| 46 | AMD1 |  |  | 3955 | -0.499 | -0.5238 | No |
| 47 | INCENP |  |  | 3985 | -0.507 | -0.5240 | No |
| 48 | CENPF |  |  | 4003 | -0.511 | -0.5217 | No |
| 49 | NEK2 |  |  | 4086 | -0.536 | -0.5318 | Yes |
| 50 | EXO1 |  |  | 4094 | -0.539 | -0.5273 | Yes |
| 51 | CCNB2 |  |  | 4103 | -0.542 | -0.5229 | Yes |
| 52 | NCL |  |  | 4171 | -0.560 | -0.5298 | Yes |
| 53 | PLK1 |  |  | 4205 | -0.569 | -0.5300 | Yes |
| 54 | BUB1 |  |  | 4246 | -0.578 | -0.5315 | Yes |
| 55 | BUB3 |  |  | 4249 | -0.580 | -0.5256 | Yes |
| 56 | SNRPD1 |  |  | 4254 | -0.582 | -0.5200 | Yes |
| 57 | MCM6 |  |  | 4273 | -0.590 | -0.5171 | Yes |
| 58 | MAP3K20 |  |  | 4308 | -0.599 | -0.5172 | Yes |
| 59 | DR1 |  |  | 4322 | -0.603 | -0.5131 | Yes |
| 60 | KIF15 |  |  | 4324 | -0.603 | -0.5068 | Yes |
| 61 | SLC12A2 |  |  | 4347 | -0.614 | -0.5044 | Yes |
| 62 | KPNA2 |  |  | 4361 | -0.621 | -0.5001 | Yes |
| 63 | KNL1 |  |  | 4367 | -0.624 | -0.4943 | Yes |
| 64 | POLA2 |  |  | 4379 | -0.627 | -0.4896 | Yes |
| 65 | PML |  |  | 4415 | -0.641 | -0.4894 | Yes |
| 66 | ODC1 |  |  | 4437 | -0.649 | -0.4865 | Yes |
| 67 | UCK2 |  |  | 4438 | -0.649 | -0.4794 | Yes |
| 68 | DDX39A |  |  | 4467 | -0.661 | -0.4776 | Yes |
| 69 | RBL1 |  |  | 4469 | -0.662 | -0.4706 | Yes |
| 70 | SLC7A5 |  |  | 4544 | -0.701 | -0.4773 | Yes |
| 71 | ATF5 |  |  | 4558 | -0.712 | -0.4721 | Yes |
| 72 | DTYMK |  |  | 4566 | -0.715 | -0.4657 | Yes |
| 73 | DBF4 |  |  | 4570 | -0.717 | -0.4585 | Yes |
| 74 | PRIM2 |  |  | 4585 | -0.728 | -0.4533 | Yes |
| 75 | PBK |  |  | 4593 | -0.730 | -0.4467 | Yes |
| 76 | CKS2 |  |  | 4600 | -0.733 | -0.4399 | Yes |
| 77 | SLC7A1 |  |  | 4614 | -0.740 | -0.4344 | Yes |
| 78 | STIL |  |  | 4615 | -0.740 | -0.4264 | Yes |
| 79 | BIRC5 |  |  | 4618 | -0.745 | -0.4187 | Yes |
| 80 | RACGAP1 |  |  | 4623 | -0.748 | -0.4113 | Yes |
| 81 | E2F1 |  |  | 4637 | -0.760 | -0.4056 | Yes |
| 82 | RPA2 |  |  | 4638 | -0.760 | -0.3973 | Yes |
| 83 | TTK |  |  | 4644 | -0.762 | -0.3900 | Yes |
| 84 | DKC1 |  |  | 4646 | -0.763 | -0.3819 | Yes |
| 85 | NUSAP1 |  |  | 4654 | -0.765 | -0.3749 | Yes |
| 86 | KPNB1 |  |  | 4681 | -0.786 | -0.3714 | Yes |
| 87 | CENPA |  |  | 4683 | -0.788 | -0.3631 | Yes |
| 88 | CDC45 |  |  | 4702 | -0.798 | -0.3579 | Yes |
| 89 | KIF4A |  |  | 4727 | -0.812 | -0.3537 | Yes |
| 90 | KIF20B |  |  | 4745 | -0.825 | -0.3480 | Yes |
| 91 | CHAF1A |  |  | 4751 | -0.828 | -0.3400 | Yes |
| 92 | CENPE |  |  | 4757 | -0.832 | -0.3319 | Yes |
| 93 | KIF11 |  |  | 4760 | -0.835 | -0.3232 | Yes |
| 94 | CHEK1 |  |  | 4764 | -0.837 | -0.3147 | Yes |
| 95 | TGFB1 |  |  | 4781 | -0.846 | -0.3086 | Yes |
| 96 | TMPO |  |  | 4790 | -0.851 | -0.3009 | Yes |
| 97 | MCM5 |  |  | 4792 | -0.853 | -0.2919 | Yes |
| 98 | CDK4 |  |  | 4810 | -0.874 | -0.2857 | Yes |
| 99 | KIF23 |  |  | 4825 | -0.891 | -0.2787 | Yes |
| 100 | MCM3 |  |  | 4834 | -0.901 | -0.2704 | Yes |
| 101 | MKI67 |  |  | 4836 | -0.902 | -0.2608 | Yes |
| 102 | PRC1 |  |  | 4846 | -0.912 | -0.2527 | Yes |
| 103 | SMC2 |  |  | 4847 | -0.912 | -0.2428 | Yes |
| 104 | ORC6 |  |  | 4865 | -0.931 | -0.2359 | Yes |
| 105 | MYC |  |  | 4883 | -0.947 | -0.2289 | Yes |
| 106 | GINS2 |  |  | 4897 | -0.962 | -0.2210 | Yes |
| 107 | TACC3 |  |  | 4900 | -0.966 | -0.2109 | Yes |
| 108 | AURKA |  |  | 4906 | -0.973 | -0.2013 | Yes |
| 109 | FBXO5 |  |  | 4910 | -0.978 | -0.1912 | Yes |
| 110 | SLC38A1 |  |  | 4971 | -1.043 | -0.1915 | Yes |
| 111 | HMMR |  |  | 4993 | -1.080 | -0.1839 | Yes |
| 112 | MAD2L1 |  |  | 5007 | -1.105 | -0.1744 | Yes |
| 113 | AURKB |  |  | 5016 | -1.118 | -0.1638 | Yes |
| 114 | KIF2C |  |  | 5028 | -1.150 | -0.1534 | Yes |
| 115 | TOP2A |  |  | 5029 | -1.152 | -0.1409 | Yes |
| 116 | MYBL2 |  |  | 5041 | -1.172 | -0.1303 | Yes |
| 117 | TPX2 |  |  | 5049 | -1.184 | -0.1188 | Yes |
| 118 | CCNA2 |  |  | 5051 | -1.186 | -0.1061 | Yes |
| 119 | NDC80 |  |  | 5055 | -1.200 | -0.0936 | Yes |
| 120 | MT2A |  |  | 5061 | -1.216 | -0.0814 | Yes |
| 121 | BRCA2 |  |  | 5069 | -1.222 | -0.0694 | Yes |
| 122 | CDC20 |  |  | 5082 | -1.249 | -0.0582 | Yes |
| 123 | UBE2C |  |  | 5106 | -1.314 | -0.0484 | Yes |
| 124 | CDC6 |  |  | 5107 | -1.314 | -0.0341 | Yes |
| 125 | CKS1B |  |  | 5121 | -1.338 | -0.0221 | Yes |
| 126 | CDK1 |  |  | 5164 | -1.512 | -0.0138 | Yes |
| 127 | TLE3 |  |  | 5206 | -1.684 | -0.0034 | Yes |
| 128 | HMGA1 |  |  | 5220 | -1.792 | 0.0136 | Yes |
Table: GSEA details [plain text format]

  

Fig 2: HALLMARK\_G2M\_CHECKPOINT: Random ES distribution      
 Gene set null distribution of ES for **HALLMARK\_G2M\_CHECKPOINT**
